# Change of serum uric acid and progression of cardiometabolic multimorbidity among middle aged and older adults: A prospective cohort study

**DOI:** 10.3389/fpubh.2022.1012223

**Published:** 2022-10-26

**Authors:** Duanhui Li, Danyang Wang, Xiaochen Dai, Yujie Ni, Xiaolin Xu

**Affiliations:** ^1^Department of Big Data in Health Science School of Public Health Center of Clinical Big Data and Analytics of The Second Affiliated Hospital, Zhejiang University School of Medicine, Hangzhou, China; ^2^Department of Health Metrics Sciences, School of Medicine, University of Washington, Seattle, WA, United States; ^3^Institute for Health Metrics and Evaluation, University of Washington, Seattle, WA, United States; ^4^Faculty of Medicine, School of Public Health, The University of Queensland, Brisbane, QLD, Australia

**Keywords:** cardiometabolic multimorbidity, diabetes mellitus, cardiovascular disease, serum uric acid, progression, prospective cohort study, hyperuricemia

## Abstract

**Background:**

Hyperuricemia is prevalent and associated with individual cardiometabolic diseases, highlighting the potential role of serum uric acid (SUA) in the development and progression of cardiometabolic multimorbidity (CMM, the coexistence of diabetes, heart disease, or stroke). This study aimed to examine the role of SUA change in the progression of CMM.

**Methods:**

This prospective cohort study used data from the China Health and Retirement Longitudinal Study, included 4,820 participants aged 45 years or above with three complete surveys at 2011 (baseline), 2015, and 2018. SUA level at survey 2011 and 2015 was used to measure SUA change as keeping or rising to hyperuricemia, and keeping or declining to non-hyperuricemia. CMM progression was defined as the first report of CMM or additional report of cardiometabolic diseases during survey 2015 and 2018. We used logistic regression models to estimate the odds ratios (ORs) and 95% confidence intervals (95% CIs) of SUA change on CMM progression.

**Results:**

During the follow-up of around 7 years, 519 (10.8%) of the participants kept or rose to hyperuricemia from survey 2011 to 2015, and 311 (6.5%) experienced CMM progression from survey 2015 to 2018. Participants who kept or rose to hyperuricemia had 1.86 (95% CI, 1.29, 2.68) increased odds of CMM progression compared with those who kept or declined to non-hyperuricemia. Specifically, keeping or rising to hyperuricemia (vs. keeping or declining to non-hyperuricemia) was associated with 2.01 times higher odds (95% CI, 1.18, 3.43) of incident diabetes and 1.67 times higher odds (OR:1.67; 95% CI, 1.15, 2.43) of incident cardiovascular diseases following diabetes.

**Conclusion:**

Keeping or rising to hyperuricemia was associated with CMM progression, particularly with incident cardiovascular diseases following diabetes. These findings suggest that monitoring SUA change may provide innovative insights into the prevention of CMM, especially in the secondary prevention of CMM (i.e., preventing further progression to cardiovascular diseases among patients with diabetes).

## Introduction

Cardiometabolic multimorbidity (CMM), the coexistence of diabetes, heart disease, or stroke (1), is the most common multimorbidity pattern (2, 3) and constitutes the major disease burden worldwide including China, particularly in the era of COVID-19 pandemic (4, 5). A pooled analysis of 91 cohort studies showed that the mortality risk of any combinations of cardiometabolic diseases was substantially greater than that of each individual diseases (1). Due to disease-disease interactions, CMM complicates the treatment regimen and greatly challenges healthcare systems configured for single diseases (6). Therefore, evidence informing strategies for prevention and management the development and progression of CMM is urgently needed.

Uric acid is an end product of purine metabolism (7) and traditionally implicated in gout and kidney stones formation (8). Several studies showed that elevated serum uric acid (SUA) was a significant risk factor for incident diabetes (9, 10) and cardiovascular diseases (CVD) mortality (11). Furthermore, UA was suggested as a potential therapeutic target for CVD (12). However, the mechanism by which SUA affecting cardiometabolic diseases is still inconclusive and few studies have ever examined the effect of SUA change. Many studies suggest an independent association between SUA and cardiovascular diseases or mortality, but the association may be explained by its complex relationship with other cardiovascular risk factors (13), for example, a study used data from Framingham cohort found that SUA level was associated with risk for incident coronary heart disease, CVD death, or all-cause death among women, but in fully adjusted multivariate Cox models, the association was no longer significant (14). Recently, rapid development in detection methods of uric acid has made it easy to acquire information on change of uric acid (15). Assessing the effect of uric acid change on CMM progression may help informing risk prediction and intervention targets for the efficient management and prevention of CMM. Using longitudinal data of representative Chinese middle-aged and elderly adults, the present study aimed to explore the association between SUA change and CMM progression.

## Methods

### Study participants

**Figure 1:**
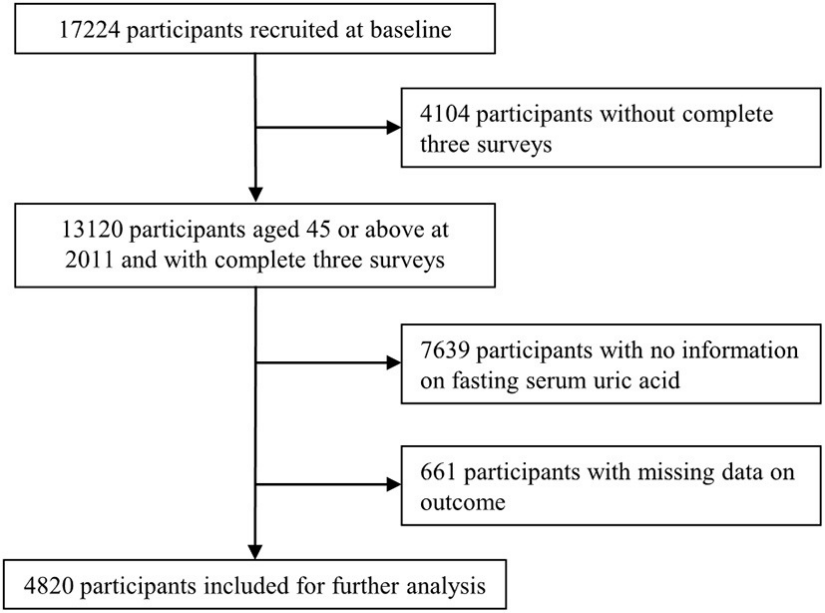
Flowchart showing the selection of participants.

The China Health and Retirement Longitudinal Study (CHARLS) is a nationally representative prospective cohort which recruited adults aged 45 or older at baseline and followed up regularly to collect information on the demographics, socioeconomics, physical and psychological health. The baseline survey (wave 1) was conducted in 2011–12, with wave 2 in 2013, wave 3 in 2015, and wave 4 in 2018. In order to ensure sample representativeness, the CHARLS baseline survey covered 150 countries/districts, 450 villages/urban communities, across the country, involving 17,708 individuals in 10,257 households, reflecting the mid-aged and older Chinese population collectively. Details of the study have been described elsewhere (16). As of writing, four surveys of 2011, 2013, 2015, 2018 were publicly available. Because blood samples were only collected at survey 2011 and 2015, we chose three surveys of 2011, 2015, 2018 for the present study. Participants aged 45 or above at 2011 and with complete information on exposures and outcomes of interest across the three surveys were included. Participants were excluded if they (a) did not complete the three selected surveys; (b) had missing data on serum uric acid at survey 2011 or 2015; (c) had missing data on diabetes, heart disease, or stroke at survey 2015 or 2018 (Figure 1).

### Assessment of SUA change

Blood samples were collected from each participant after fasting overnight and the methods of laboratory assay for the SUA have been described elsewhere (17). Hyperuricemia was defined as SUA level >7 mg/dl in men and >6 mg/dl in women (18). SUA levels at survey 2011 and 2015 was used to measure SUA change. In the present study, SUA change had two forms: (1) the continuous change of SUA concentration between survey 2011 and 2015; (2) the categorical variable of keeping or rising to hyperuricemia, and keeping or declining to non-hyperuricemia from 2011 to 2015.

### Assessment of CMM and CMM progression

At each survey, participants were asked whether or not a doctor has told them they had diabetes, heart disease (including heart attack, coronary heart disease, angina, congestive heart failure, or other heart problems) or stroke, to which they could respond “yes” or “no.” Where “Yes” was reported, the timing of diagnoses, current medications and treatments for each specific condition were further collected. Thus, the self-report diagnosis can be validated by diagnosis time or medications. CMM referred to the coexistence of two or more diseases from diabetes, heart disease, and stroke. From survey 2015 to 2018, the first report of individual diseases or CMM after survey 2015 was considered as incidence. The progression of CMM, as defined in previous studies (2), indicated the first report of CMM or additional report of cardiometabolic disease among participants who were already with CMM after survey 2015 (Table 1).

**Table 1:** Definition of progression of CMM.

**No. of cardiometabolic diseases**			**CMM progression**
**Survey 2015**	**Survey 2018**	**Change of No**.	
0	0	0 to 0, stable	**×**
0	1	0–1	**×**
0	2	0–2	**√**
0	3	0–3	**√**
1	1	1 to 1, stable	**×**
1	2	1–2	**√**
1	3	1–3	**√**
2	2	2 stable	**×**
2	3	2–3	**√**
3	3	3–3, stable	**×**

√indicates situations which can be considered as CMM progression. X indicates situations which can not be considered as CMM progression.

### Assessment of covariates

Covariates were assessed based on self-reported data at survey 2011 (baseline). There were four groups of covariates: demographic information including age and sex (male, female); health behaviors including physical activity (inactive, active), alcohol consumption (less than weekly drinking, weekly drinking or more), smoking status (never, ever), body mass index (BMI, weight divided by the square of height), and history of other chronic conditions including systolic blood pressure (SBP), liver disease (yes, no), kidney disease (yes, no), and dyslipidaemia (yes, no); and other serum biomarkers including high-density lipoprotein (HDL, < 40 mg/dl, ≥40 mg/dl), low-density lipoprotein (LDL, < 160 mg/dl, ≥160 mg/dl), total cholesterol (TC, < 240 mg/dl, ≥240 mg/dl), fasting blood glucose (FBG, < 126 mg/dl, ≥126 mg/dl). Notably, BMI was based on physically measured height and weight and categorized as underweight (< 18.5 kg/m^2^), healthy (18.5–24.9 kg/m^2^), overweight or obese (≥25 kg/m^2^). Details can be seen in Table 2.

**Table 2:** Baseline characteristics (2011) according to serum uric acid change.

**Characteristics**	**Serum uric acid**		***P***
	**Keeping or rising to hyperuricemia (*n* = 4,301)**	**Keeping or declining to non-hyperuricemia (*n* = 519)**	
Age, years (median [IQR])	59.0 [53.0, 67.0]	58.0 [52.0, 64.0]	< 0.001
Sex (%)			0.001
Male	1,882 (43.8)	266 (51.3)	
Female	2,419 (56.2)	253 (48.7)	
SBP, mmHg (median [IQR])	135.2 [122.5, 148.7]	128.3 [116.7, 142.0]	< 0.001
Body mass index, kg/m^2^ (%)			< 0.001
Underweight (< 18.5)	213 (5.7)	9 (2.0)	
Healthy (18.5–23.9)	2,017 (53.6)	183 (40.5)	
Overweight (≥24)	1,534 (40.8)	260 (57.5)	
Marital status (%)			0.854
Married	3,861 (89.8)	464 (89.4)	
Separated/divorced/widowed/never married	440 (10.2)	55 (10.6)	
Education (%)			0.058
less than upper secondary education	2,092 (48.6)	229 (44.1)	
Upper secondary & vocational training & tertiary education	2,209 (51.4)	290 (55.9)	
Smoking (%)			0.762
Never	3,035 (71.4)	364 (70.7)	
Ever/now	1,214 (28.6)	151 (29.3)	
Alcohol drinking (%)			0.032
Less than weekly drinking	3,410 (83.7)	389 (79.7)	
Weekly drinking or more	666 (16.3)	99 (20.3)	
Physical activity^*^ (%)			0.242
Inactive	572 (31.4)	82 (36.9)	
Active	1,249 (68.6)	140 (63.1)	
Dyslipidaemia (%)			< 0.001
No	3,779 (89.2)	420 (82.5)	
Yes	458 (10.8)	89 (17.5)	
Liver disease (%)			1.000
No	4,126 (96.3)	498 (96.3)	
Yes	160 (3.7)	19 (3.7)	
Kidney disease (%)			0.847
No	4,019 (93.8)	484 (93.6)	
Yes	265 (6.2)	33 (6.4)	
HDL (%)			< 0.001
< 40 mg/dl	3,324 (77.3)	327 (63.0)	
≥3. mg/dl	977 (22.7)	192 (37.0)	
LDL (%)			0.007
≤ .00 mg/dl	3,873 (90.0)	447 (86.1)	
>160 mg/dl	428 (10.0)	72 (13.9)	
FBG (%)			0.001
< 126 mg/dl	3,803 (88.4)	434 (83.6)	
≥3.6 mg/dl	498 (11.6)	85 (16.4)	
TG (%)			< 0.001
< 240 mg/dl	3,839 (89.3)	436 (84.0)	
≥4.0 mg/dl	462 (10.7)	83 (16.0)	

SBP, Systolic Blood Pressure; HDL, High Density Liptein Cholesterol; LDL, Low Density Liptein Cholesterol; FBG, Fasting Blood Glucose; TG, Triglyceride.

* In CHARLS wave 1–3 (2011, 2013, 2015), the questions on physical activity were only asked to a random subsample of half the sample, and those who were not selected were assigned a special missing value.

### Statistical analysis

Baseline characteristics were described by SUA change (i.e., keeping or rising to hyperuricemia, keeping or declining to non-hyperuricemia). Mean and standard deviation, or frequency and percentages were used to describe continuous or categorical variables, respectively. Differences between groups were examined using *t*-tests or chi-squared tests.

#### Main analysis

The primary aim of this study was to explore the role of SUA change on CMM progression. We firstly used multivariable logistic regression models (adjusted for age, sex, and health behaviors) to examine the associations of keeping hyperuricemia (vs. declining to non-hyperuricemia), and rising to hyperuricemia (vs. keeping non-hyperuricemia) with CMM progression among participants with or without hyperuricemia at baseline, respectively. Secondly, we further examined the effect of four categorized group of SUA change with “keeping non-hyperuricemia” as reference in the whole population. Thirdly, we aggregated the exposure (keeping or rising to hyperuricemia vs. keeping or declining to non-hyperuricemia) and investigated their association with CMM progression. Furthermore, we analyzed the change of SUA as a continuous variable and used restricted cubic splines with five knots to visualize the shape of the association. Wald chi-square tests were used to test whether the null hypothesis of linear association can be rejected. Lastly, we restricted population to those without CMM at survey 2015 and used multinomial logistic regression models to examine the association of the SUA change with different transitions (Supplementary Figure 1) of cardiometabolic diseases (19): (1) transition from no conditions to incident diabetes only, (2) transition from no conditions to incident CVD only, (3) transition from no conditions to CMM, (4) cardiovascular diseases (heart disease or stroke) followed-by diabetes, (5) diabetes followed-by cardiovascular diseases. In this multinomial analysis, participants with none of the three cardiometabolic diseases from survey 2015 to 2018 were the reference group. All the analyses above reported associations as odds ratios (ORs) with 95% confidence intervals (CIs). For each analysis, models adjusting for age at baseline, sex, BMI, SBP and behaviors.

#### Subgroup analysis and sensitivity analysis

We performed subgroup analysis stratified by age, sex, BMI, smoking, alcohol, and physical activates, respectively, to examine the variations in the associations of the aggregated SUA change with CMM progression. To check the robustness of our main findings, we run models with additional adjustment for history of chronic conditions and other serum biomarkers to examine the association between the aggregated SUA change and progression of CMM.

All analysis was performed using SAS (version 9.4, SAS Institute Inc.) and R (version 4.0.5). All statistical tests were two-side, and *P* < 0.05 was considered to be statistically significant.

## Results

### Characteristics of participants

Of the 4,820 participants included, participants who kept or rose to hyperuricemia were more likely to be older, male, overweight, consume alcohol more frequently, have dyslipidaemia, have higher SBP, LDL and lower HDL, TG, FBG (Table 2).

### Associations of SUA change with CMM progression

**Figure 2:**
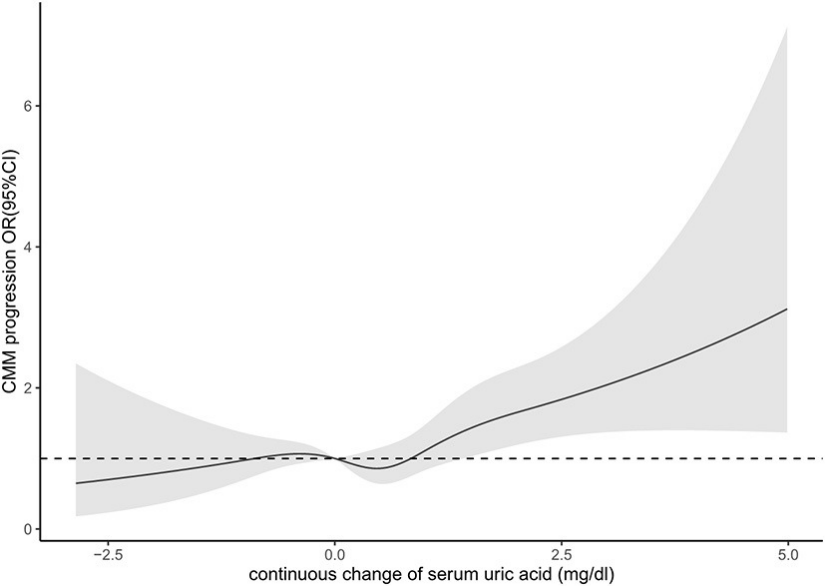
Association between continuous change of serum uric acid and progression of cardiometabolic multimorbidity.

From survey 2011 to 2015, 88 (1.8%) of the participants kept hyperuricemia and 382 (7.9%) rose to hyperuricemia, while 4,213 (87.4%) kept non-hyperuricemia and 137 (2.8%) declined to non-hyperuricemia (Table 3). Over approximate 3-year follow-up, 311 (6.5%) participants experienced CMM progression. Among participants without hyperuricemia at baseline, rising to hyperuricemia (vs. keeping non-hyperuricemia) was associated with 1.98 elevated odds (95% CI, 1.32, 2.95, *P* < 0.001) of CMM progression. Among participants with hyperuricemia at baseline, keeping hyperuricemia (vs. declining to non-hyperuricemia) was associated with 2.46 elevated odds (OR, 2.46; 95% CI, 0.53, 11.42, *P* = 0.25) of CMM progression. In terms of the four categorized group with keeping non-hyperuricemia as reference, rising to hyperuricemia was significantly associated with higher odds (OR, 2.00; 95% CI, 1.34, 2.98, *P* = 0.01) of CMM progression in the whole population. With For participants who kept or rose to hyperuricemia, the odds of CMM progression was 1.86 (95% CI, 1.29, 2.68, *P* < 0.001) times higher than participants who kept or declined to non-hyperuricemia. In model with continuous change of SUA as exposure, we found each unit increase in SUA was associated with 1.23 times higher odds (95%CI, 1.08, 1.39, *P* = 0.001) of CMM progression. According to the restricted cubic splines, there was no evidence demonstrate that nonlinear association between SUA concentration change and CMM progression (*P* for non-linear trend = 0.20) (Figure 2).

**Table 3:** Associations between change of serum uric acid and progression of cardiometabolic multimorbidity.

**Populations**	**Exposure**	**No. and % of case**	**ORs (95% CIs)**	***P*-value**
Participants with hyperuricemia at baseline	Declining to non-hyperuricemia (*n* = 137)	7 (7.95%)	1.00 (ref)	0.248
(*n* = 225)	Keeping hyperuricemia (*n* = 88)	14 (10.22%)	2.46 (0.53, 11.42)	
Participants without hyperuricemia at baseline	Keeping non-hyperuricemia (*n* = 4,213)	249 (5.91%)	1.00 (ref)	< 0.001
(*n* = 4,595)	Rising to hyperuricemia (*n* = 382)	41 (10.73%)	1.98 (1.32, 2.95)	
The whole population (*n* = 4,820)	Declining to non-hyperuricemia (*n* = 137)	7 (7.95%)	0.63 (0.19, 2.13)	0.21
	Keeping hyperuricemia (*n* = 88)	14 (10.22%)	1.37 (0.65, 2.90)	0.58
	Keeping non-hyperuricemia (*n* = 4,213)	249 (5.91%)	1.00 (ref)	
	Rising to hyperuricemia (*n* = 382)	41 (10.73%)	2.00 (1.34, 2.98)	0.01
The whole population (*n* = 4,820)	Keeping or declining to non-hyperuricemia (*n* = 4,301)	256 (5.95%)	1.00 (ref)	< 0.001
	Keeping or rising to hyperuricemia (*n* = 519)	55 (10.60%)	1.86 (1.29, 2.68)	
The whole population (*n* = 4,820)	Continuous change of SUA	311 (6.45%)	1.23 (1.08, 1.39)	0.001

Case here indicates participants experiencing progression of cardiometabolic multimorbidity. Details on the definition of progression can be seen in Table 1. Model: Adjust for age, sex, and health behaviors (smoking, alcohol consumption, physical activity).

### Associations of SUA change with transitions of cardiometabolic diseases

Of the 4,576 participants without CMM at survey 2015, 123 (2.7%) participants developed diabetes only from survey 2015 to 2018, 341 (7.5%) developed CVD only, 57 (1.2%) developed CMM, 306 (6.7%) developed cardiovascular diseases following diabetes and 76 (1.7%) developed diabetes following cardiovascular diseases. Compared to participants who kept or declined to non-hyperuricemia, participants who kept or rose to hyperuricemia had 2.04 higher odds (95% CI, 1.20, 3.47, *P* = 0.009) of developing diabetes only, and 1.67 higher odds (95% CI, 1.15, 2.43, *P* = 0.007) of developing cardiovascular diseases following diabetes. We found no significant associations between SUA change and developing CVD only, CMM, or diabetes following cardiovascular diseases (Table 4).

**Table 4:** Multinomial logistic regression analysis of the associations between change of serum uric acid and different transitions of cardiometabolic diseases.

**Transitions of cardiometabolic diseases**		**The population without CMM at survey 2015 (*****n*** = **4,576)**	
		**Keeping or declining to non-hyperuricemia (*n* = 4,096)**	**Keeping or Rising to hyperuricemia (*n* = 480)**
No conditions to diabetes (*n* = 123)	Observed prevalence	2.39% (98/4,096)	5.21% (25/480)
	OR (95% CI)	1.00 (ref)	2.04 (1.20, 3.47)
	*P*-value	0.009	
No conditions to CVD (*n* = 341)	Observed prevalence	7.54% (309/4,096)	6.66% (32/480)
	OR (95% CI)	1.00 (ref)	0.97 (0.64, 1.49)
	*P*-value	0.905	
No conditions to CMM (*n* = 57)	Observed prevalence	1.12% (46/4,096)	2.29% (11/480)
	OR (95% CI)	1.00 (ref)	2.02 (0.98, 4.18)
	*P*-value	0.057	
Diabetes to CVD (*n* = 306)	Observed prevalence	6.15% (252/4,096)	11.25% (54/480)
	OR (95% CI)	1.00 (ref)	1.67 (1.15, 2.43)
	*P*-value	0.007	
CVD to diabetes (*n* = 76)	Observed prevalence	1.59% (65/4,096)	2.29% (11/480)
	OR (95% CI)	1.00 (ref)	1.51 (0.74, 3.09)
	*P*-value	0.257	

Models were adjusted for age, sex, and health behaviors (smoking, alcohol consumption, physical activity). CMM, cardiometabolic multimorbidity; CVD, cardiovascular disease, the diagnosis of either heart disease or stroke.

### Subgroup analysis and sensitivity analysis

The subgroup analysis and sensitivity analysis were consistent with our main results. However, we also observed variations in the associations of the aggregated SUA change with CMM progression in different subgroups (Supplementary Figure 1). For example, the ORs of the aggregated SUA change on CMM progression was larger in males than females. In models with additional adjustment for history of chronic conditions and serum biomarkers (Supplementary Tables 1, 2), we found that keeping or rising to hyperuricemia (vs. keeping or declining to non-hyperuricemia) was significantly associated with the progression of CMM (OR, 1.77; 95% CI, 1.18, 2.64, *P* = 0.006).

## Discussion

We analyzed 4,820 middle-aged or elderly Chinese adults and found keeping or rising to hyperuricemia was associated with higher odds of CMM progression. Specifically, keeping or rising to hyperuricemia was associated with higher odds of incident cardiovascular diseases among patients with diabetes.

### Interpretation and comparison with other studies

To our knowledge, this is the first study to delineate the role of SUA change in the progression of diabetes, heart disease, stroke, and CMM. In our study, keeping or rising to hyperuricemia was associated with diabetes (OR, 1.86; 95% CI, 1.29, 2.68, *P* < 0.001), compared with keeping or declining to non-hyperuricemia; but showed no effects on heart disease (OR, 1.05; 95% CI, 0.62, 1.78, *P* = 0.86) and stroke (OR, 0.88; 95% CI, 0.45, 1.73, *P* = 0.68), which is consistent with prior studies. For example, a meta-analysis (42,834 participants) suggests that SUA level is positively associated with the development of type 2 diabetes regardless of various study characteristics (18), and evidence from the British Regional Heart Study, which included middle-aged 7,735 men (average 16.8 years follow-up), found that SUA was strongly correlated with many CVD risk factors and was positively associated with risk for fatal and non-fatal CHD events. However, after full adjustment for potentially confounding clinical factors, this relationship was no longer significant (20). However, as for CVD, other studies demonstrated the associations between SUA and CVD, for example, a prospective study, found that compared with individuals in the bottom third of baseline measurements of serum uric acid in the Reykjavik study, those in the top third had an age- and sex-adjusted odds ratio for CHD of 1.39 (95% CI, 1.23–1.58) (20). Evidence from the Third National Health and Nutrition Examination Survey revealed an increased risk of CV mortality with increasing SUA levels, hazard ratio (95% CI) per 59.5 mmol/l of SUA was 1.32 (1.25–1.38), and remained 1.15 (1.08–1.21) even after adjusted for demographic factors, comorbidities and other risk factors (21). The role of SUA as a risk factor for developing cardiovascular disease is controversial (22), and the association between SUA and CVD may be explained by its complex relationship with other cardiovascular risk factors (23).

Besides, evidence from previous studies indicated that high uric acid is independently associated with an increased risk of new-onset CVD in patients with diabetes (21, 22). Which is consistent with our finding that keeping or rising to hyperuricemia was significantly associated with the incidence of cardiovascular diseases among patients with diabetes (OR, 1.67; 95% CI, 1.15, 2.44, *P* = 0.007). Our data for incidence and transition of diabetes to heart disease added to new evidence by suggesting that keeping or rising to hyperuricemia is strongly linked to cardiometabolic multimorbidity, particularly among those who had already diagnosed with diabetes.

The findings reflected that cardiometabolic conditions are not simply co-occurring through chance. Zemedikun et al. used a combination of cluster analysis and data mining techniques identified that diabetes might be the epicenter of disease clusters for multimorbidity (23). There is evidence that certain conditions are more likely to cluster due to shared pathological pathways or networks, whereby the onset of one condition increases the risk of another (24), previous studies showed that associations of diabetes with chronic disease outcomes are largely independent of major cardiovascular risk factors (25, 26) and diabetes is a well-established risk factor for heart disease and stroke. Although the underlying mechanisms for higher risks of CVD among participants with diabetes remain unclear, previous studies have suggested hyperuricemia in patients with diabetes causes incremental variations in uric acid levels over time, thus increasing oxidative stress and generating free radicals, which contribute to endothelial dysfunction and RAAS activation, ultimately leading to CVD (13, 21). But studies also suggested that the apparent association might be explained that SUA caused CVD is primarily a function of SUA being strongly collinear with established CVD risk factors (14, 27).

Cardiometabolic multimorbidity, one of the most common multimorbidity pattern, could accelerate the development and progression of other conditions like mental or musculoskeletal disorders (28). Exploring the association between SUA change and the progression of CMM may help us to develop methods to interrupt the progression; furthermore, it can also provide evidence for secondary prevention of developing other diseases. Hence, clinicians should pay attention to the SUA levels and changes over time, particularly among patients with diabetes. Besides, studies also suggested that some lifestyle factors (e.g., smoke, alcohol consumption, less healthy dietary habits) played important roles in the progression of CMM (29, 30); therefore, lifestyle modifications those targeted for keeping or declining serum uric acid level and the development of cardiometabolic diseases should be emphasized especially among patients with diabetes.

### Strengths and limitations

Using representative sample of Chinese middle-aged and elderly adults and prospective study design, that study may provide reliable associations between uric acid and cardiometabolic diseases. In addition, we measured the longitudinal change of SUA, instead of cross-sectional status, and comprehensively explored the associations between SUA change with different transition patterns of cardiometabolic diseases, adding novel insights into the role of SUA in the networks of cardiometabolic diseases. This study also has several limitations. First, most variables of interest were self-reported and thus susceptible to reporting errors. Second, CMM progression was a chronic process, and 3-year follow-up may not long enough to capture the long-term progression. Third, information about medications affecting SUA change, such as diuretics and beta blockers were potential confounders and not adjusted in models because of limited data availability. Forth, we used frequency, instead of amount (e.g., volume or weight) to assess drinking behavior, which may be not accurate enough. Finally, due to the small sample size and short-time period, the progression from no conditions to diabetes and then to CVD, and the progression from no conditions to CVD and then to diabetes cannot be captured; which guaranteed further studies.

## Conclusions

Keeping or rising to hyperuricemia was associated with CMM progression, particularly associated with higher odds of developing CVD after diabetes. Our study highlighted the role of SUA change in interpreting the mechanisms for the progression of CMM, and the need for healthcare professionals to monitor SUA change for efficient management of CMM, especially in the secondary prevention of diabetes (i.e., preventing further progression to CMM).

## Data availability statement

The raw data supporting the conclusions of this article will be made available by the authors, without undue reservation.

## Ethics statement

The studies involving human participants were reviewed and approved by Biomedical Ethics Committee of Peking University (No. IRB00001052–11015). The patients/participants provided their written informed consent to participate in this study.

## Author contributions

XX conceptualized the study, supervised the whole project, had full access to all the data in the study, and had final responsibility for the decision to submit for publication. DL, DW, and XX made the analysis plan. DL conducted the statistical analyses. DL and DW wrote the initial draft of the manuscript. YN and DW verified the underlying data. XD provided valuable support on statistical methods, drafting, and revisions of the manuscript. All authors contributed to the article and approved the final manuscript.

## Conflict of interest

The authors declare that the research was conducted in the absence of any commercial or financial relationships that could be construed as a potential conflict of interest.

## Publisher's note

All claims expressed in this article are solely those of the authors and do not necessarily represent those of their affiliated organizations, or those of the publisher, the editors and the reviewers. Any product that may be evaluated in this article, or claim that may be made by its manufacturer, is not guaranteed or endorsed by the publisher.
