# Supplementary material for: Change of serum uric acid and progression of cardiometabolic multimorbidity among middle aged and older adults: A prospective cohort study
Source: Front Public Health. 2022 Oct 26;10:1012223. doi: 10.3389/fpubh.2022.1012223 (PMC9644181; doi:10.3389/fpubh.2022.1012223)

**Figure S1.** Subgroup analysis of association between change of serum uric acid and progression of cardiometabolic multimorbidity


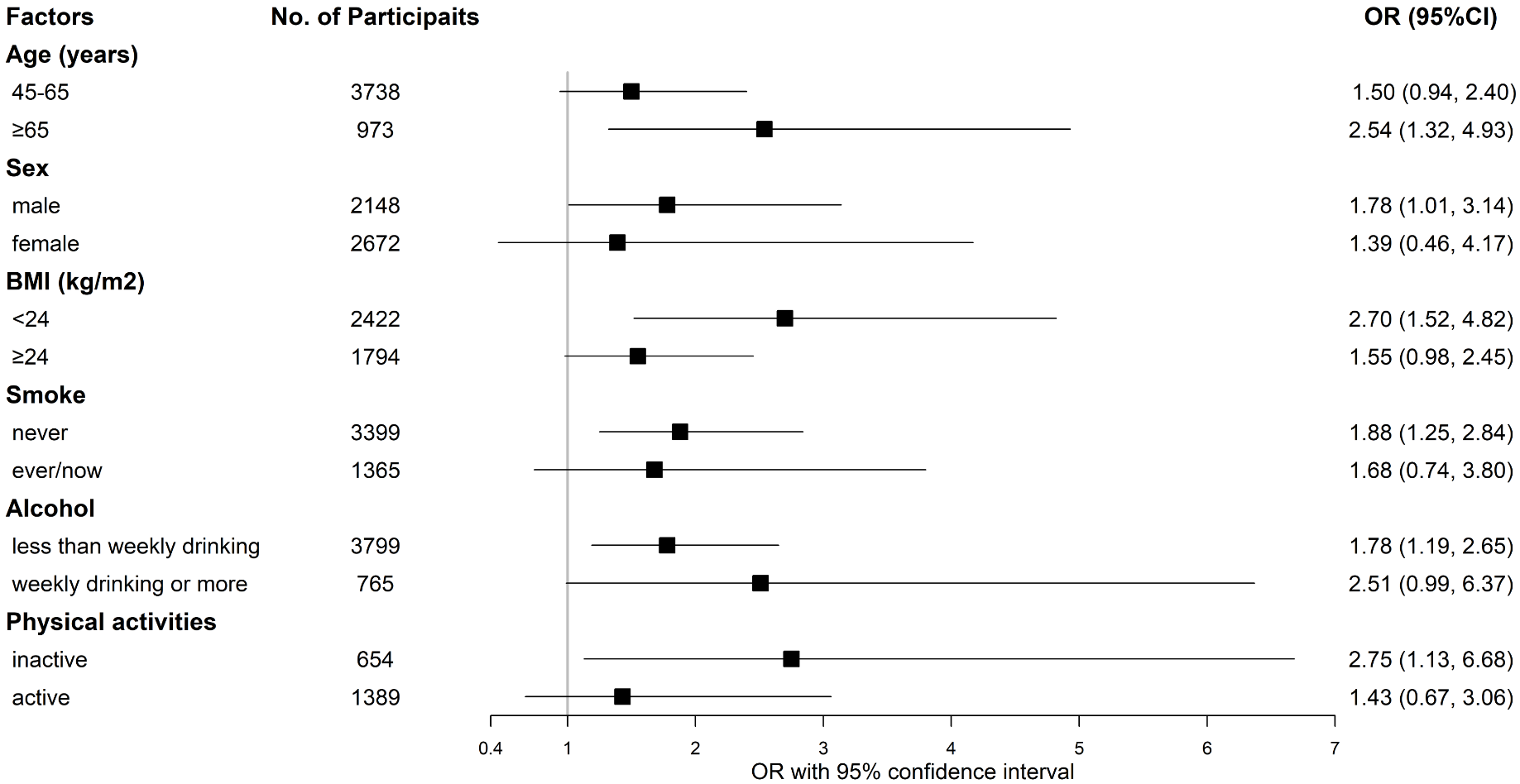

Supplement: Supplementary file 1 [file Table_1.DOCX]
