# Supplementary material for: Change of serum uric acid and progression of cardiometabolic multimorbidity among middle aged and older adults: A prospective cohort study
Source: Front Public Health. 2022 Oct 26;10:1012223. doi: 10.3389/fpubh.2022.1012223 (PMC9644181; doi:10.3389/fpubh.2022.1012223)
Supplement: Supplementary file 2 [file Table_2.DOCX]

**Table S1.** Associations between change of serum uric acid and progression of cardiometabolic multimorbidity

| **Populations** | **Exposure** | **No. and % of case** | **ORs (95% CIs)** |
| --- | --- | --- | --- |
| The whole population  (n=4820) | Keeping or declining to non-hyperuricemia (n= 4301) | 256 (5.95%) | 1.00 (ref) |
|  | Keeping or Rising to hyperuricemia (n=519) | 55 (10.60%) | 1.77 (1.18, 2.64) |
| Participants with hyperuricemia at baseline (n=225) | Declining to non-hyperuricemia (n=137) | 7 (7.95%) | 25.53 (1.04, 629.00) |
|  | Keeping hyperuricemia (n=88) | 14 (10.22%) | 2.46 (0.53, 11.42) |
| Participants without hyperuricemia at baseline (n=4595) | Keeping non-hyperuricemia (n=4213) | 249 (5.91%) | 1.00 (ref) |
|  | Rising to hyperuricemia (n=382) | 41 (10.73%) | 2.00 (1.29, 3.11) |

Note: Case here indicates participants experiencing progression of cardiometabolic multimorbidity. Details on the definition of progression can be seen in Table 1.

Models were adjusted for age, sex, socioeconomic factors, health behaviours, history of chronic conditions and serum biomarkers.
