# Supplementary material for: Change of serum uric acid and progression of cardiometabolic multimorbidity among middle aged and older adults: A prospective cohort study
Source: Front Public Health. 2022 Oct 26;10:1012223. doi: 10.3389/fpubh.2022.1012223 (PMC9644181; doi:10.3389/fpubh.2022.1012223)
Supplement: Supplementary file 3 [file Table_3.DOCX]

**Table S2.** Multinomial logistic regression analysis of the associations between change of serum uric acid and transitions of cardiometabolic diseases

| **Transitions of cardiometabolic diseases** |  | **The population without CMM at survey 2015 (N=4576)** | |
| --- | --- | --- | --- |
|  |  | **Keeping or declining to non-hyperuricemia (n=4096)** | **Keeping or Rising to hyperuricemia (n=480)** |
| **No conditions to Diabetes (n = 123)** | Observed prevalence | 2.39% (98/4096) | 5.21% (25/480) |
|  | OR (95%CI) | 1.00 (ref) | 2.07 (1.19, 3.60) |
| **No conditions to CVD (n=341)** | Observed prevalence | 7.54% (309/4096) | 6.66% (32/480) |
|  | OR (95%CI) | 1.00 (ref) | 1.00 (0.65, 1.55) |
| **No conditions to CMM (n=57)** | Observed prevalence | 1.12% (46/4096) | 2.29% (11/480) |
|  | OR (95%CI) | 1.00 (ref) | 1.83 (0.87, 3.85) |
| **Diabetes to CVD (n=306)** | Observed prevalence | 6.15% (252/4096) | 11.25% (54/480) |
|  | OR (95%CI) | 1.00 (ref) | 1.63 (1.04, 2.55) |
| **CVD to Diabetes (n=76)** | Observed prevalence | 1.59% (65/4096) | 2.29% (11/480) |
|  | OR (95%CI) | 1.00 (ref) | 1.20 (0.56, 2.56) |

Note: Models were adjusted for age, sex, socioeconomic factors, health behaviours, history of chronic conditions and serum biomarkers. CMM: cardiometabolic multimorbidity, CVD: cardiovascular disease, the diagnosis of either heart disease or stroke or both.
